# Altered Proteome of *Burkholderia pseudomallei* Colony Variants Induced by Exposure to Human Lung Epithelial Cells

**DOI:** 10.1371/journal.pone.0127398

**Published:** 2015-05-21

**Authors:** Anis Rageh Al-Maleki, Vanitha Mariappan, Kumutha Malar Vellasamy, Sun Tee Tay, Jamuna Vadivelu

**Affiliations:** Tropical Infectious Disease Research and Education Center (TIDREC), Department of Medical Microbiology, Faculty of Medicine, University of Malaya, Kuala Lumpur, Malaysia; University of Catania, ITALY

## Abstract

*Burkholderia pseudomallei* primary diagnostic cultures demonstrate colony morphology variation associated with expression of virulence and adaptation proteins. This study aims to examine the ability of *B*. *pseudomallei* colony variants (wild type [WT] and small colony variant [SCV]) to survive and replicate intracellularly in A549 cells and to identify the alterations in the protein expression of these variants, post-exposure to the A549 cells. Intracellular survival and cytotoxicity assays were performed followed by proteomics analysis using two-dimensional gel electrophoresis. *B*. *pseudomallei* SCV survive longer than the WT. During post-exposure, among 259 and 260 protein spots of SCV and WT, respectively, 19 were differentially expressed. Among SCV post-exposure up-regulated proteins, glyceraldehyde 3-phosphate dehydrogenase, fructose-bisphosphate aldolase (CbbA) and betaine aldehyde dehydrogenase were associated with adhesion and virulence. Among the down-regulated proteins, enolase (Eno) is implicated in adhesion and virulence. Additionally, post-exposure expression profiles of both variants were compared with pre-exposure. In WT pre- vs post-exposure, 36 proteins were differentially expressed. Of the up-regulated proteins, translocator protein, Eno, nucleoside diphosphate kinase (Ndk), ferritin Dps-family DNA binding protein and peptidyl-prolyl cis-trans isomerase B were implicated in invasion and virulence. In SCV pre- vs post-exposure, 27 proteins were differentially expressed. Among the up-regulated proteins, flagellin, Eno, CbbA, Ndk and phenylacetate-coenzyme A ligase have similarly been implicated in adhesion, invasion. Protein profiles differences post-exposure provide insights into association between morphotypic and phenotypic characteristics of colony variants, strengthening the role of *B*. *pseudomallei* morphotypes in pathogenesis of melioidosis.

## Introduction

Small colony variants (SCVs) are subpopulation of bacteria characterised by slower growth compared with their WT. It was first described in *Eberthella typhosa* and has atypical phenotypic and pathogenic characteristics [1]. Since then, interest on the SCVs has increased drastically where intensive studies have been reported [2–4]. The SCVs are known for their ability to resist antibiotics, remain persistent in mammalian cells and cause latent or recurrent infections in the infected host. Several pathogenic bacteria have been reported to produce SCV including, *Burkholderia cepacia* [2], *Pseudomonas aeruginosa* [5], *Salmonella* [4], *Vibrio cholera* [6], *Brucella melitensis* [7], *Escherichia coli* [8], *Serratia marcescens* [9], *Staphylococcus aureus* [10], and *Staphylococcus epidermidis* [11].

Among these pathogens, *B*. *pseudomallei*, the causative agent of melioidosis, was also found to differentiate into colony morphotypes which undergoes variation accounting up to 8.3%, in the primary cultures with one or more colony morphotypes that could reversibly change from one morphotype to another (morphotype switching) [12]. This organism has the ability to adhere to, invade, survive, persist and spread from cell-to-cell in the host [13]. As an invasive pathogenic bacteria, *B*. *pseudomallei* was also found to be resistant to several antibiotics and can survive in harsh environments [13]. In addition, *B*. *pseudomallei* is known for prolonged latency up to many years past initial infection [14] and relapse of infection with the same strain is also common despite proper and prolonged antibiotic treatment [15]. Changes in the morphotypic and phenotypic characteristics of *B*. *pseudomallei* occur when the bacteria is grown in an *in vitro* condition under different environmental parameters including starvation, iron limitation, different growth temperature, and following adaptation in experimental infection models [12, 16]. The morphologic variation is also believed to be associated with changes in the phenotypic characteristic including intracellular persistence and replication as well as alteration in expression of a range of putative virulence factors, production of extracellular enzymes, biofilm formation, flagella and also in the bacterial length [12].

Using an experimental melioidosis mouse model, Chantratita and co-workers (2007) demonstrated switching of *B*. *pseudomallei* colony morphotypes in response to stress. This adaptation process involves altered expression of surface determinants and interactions with epithelial cells and macrophages *in vitro* as well as persistence *in vivo* [12]. Additionally, Ramli *et al*., (2012) [17] also demonstrated that SCVs displayed significantly greater capacity for biofilm formation and were reduced in the ability to kill *Caenorhabditis elegans*. These studies indicate that the SCVs are able to persist extracellularly in biofilm but remain inert compared with the WT as they are shielded on a matrix, contributing to the reduced virulence.

In the previous study, using invasion and plaque formation assays, we have demonstrated that the WT was significantly more efficient than the SCV in invading and spreading from cell-to-cell in the A549 cells. In contrast, adherence assay showed that the percentage of adherence of SCV to A549 cells was found to be significantly higher than the WT [18]. Additionally, the comparative protein profile of WT and SCV in laboratory culture [before exposure to human lung epithelial cells (A549)] indicated that the differentially expressed proteins between both variants were predicted to be involved in virulence and pathogenesis due to the important roles associated with adhesion, invasion, intracellular survival and persistence in other pathogenic bacteria [18]. Further investigation on modulation of these virulence factors both in the WT and SCV during interaction with the host cells may provide an insight into the pathogenesis of these colony variants. It has also been reported that *B*. *pseudomallei* isogenic strains obtained from parental type (by starvation stress) showed a marked increase in intracellular replication fitness after 8 h of incubation [12]. However, in this study, we performed a comparative investigation on the intracellular survival abilities of *B*. *pseudomallei* WT and SCV morphotypes for 12 hours post infection to A549 cells. We also performed a comparative proteome analysis to identify differentially expressed proteins of *B*. *pseudomallei* WT and SCV upon exposure to A549. Additionally, we have performed a comparative proteome analysis of the differentially expressed proteins of both WT and SCV post-exposure to the A549 with the differentially expressed proteins of both the variants under the pre-exposure condition, which was previously reported [18]. This may provide an insight into the changes in expression of proteins that occur in the colony variants before and after infection, which in turn may aid to increase our knowledge on the virulence and pathogenesis of *B*. *pseudomallei* infection.

## Materials and Methods

### Ethics statement

In this study, ethics approval was not required since no human participant was involved. *B*. *pseudomallei* strains used in this study were obtained from the bacterial archival collection of clinical isolates available at Department of Medical Microbiology, University of Malaya. This study has an Institutional Biosafety Committee approval.

### Bacterial culture and identification

The *B*. *pseudomallei*, strain UMC074S (SCV), were differentiated from the parental isolate, UMC074L (WT), which was obtained from a melioidosis patient admitted to University Malaya Medical Centre (UMMC). Bacterial Isolation and growth condition methods were previously described [17]. Furthermore, WT and SCV morphotypes were identified by their ability to grow aerobically at 37°C on the *B*. *pseudomallei* selective media, Ashdown agar. SCVs were differentiated from the WT by their morphology and the time required for growth. WT produces clearly visible colonies within 24 hours, however, SCV produce small colonies within 48 hours. Both colonies of WT and SCV were pale purple in colour. WT produced convex, opaque and circular colonies with rough centers and smooth outer edge, and diameters of more than 5 mm whereas, SCV produced colonies with smooth centers and outer edge, but less than 2 mm in diameters. In addition, the isolates were characterised using analytical profile index (API 20NE) test (Bio-Merieux, France) and an in-house PCR assay specific for *B*. *pseudomallei* [19]. The colony variants were grown overnight on nutrient agar (NA) at 37°C and a single colony was resuspended into 10 ml Luria Bertani (LB) broth. The bacterial culture was then incubated overnight (16 hours) at 37°C in a shaking condition at 180 rpm. The bacterial culture was diluted to 1:100 in fresh LB broth and grown to mid-log phase. The culture was centrifuged at 14000 ×*g* at 4°C for 15 minutes and the pellet was incubated at 37°C in Roswell Park Memorial Institute (RPMI) medium for 30 minutes before being exposed to the lung epithelial cell line (A549).

### Cell lines

A549 cells (ATCC, No. CCL-185, Maryland, USA, passage 10–20 after defrosting from liquid nitrogen) were used in this study. Roswell Park Memorial Institute 1640 (RPMI1640) medium (Sigma Chemical, St Louis, USA) containing 10% heat inactivated fetal bovine serum (FBS) (Gibco BRL, Gland Island, NY) and 2 mM L-glutamine (Sigma Chemical, St Louis, USA) was used to culture and maintain the A549 cells. In a humidified incubator, the cells were cultured for 24 hours at 37°C under an atmosphere of 5% CO_2_ for all experiments.

### Intracellular survival assay

Intracellular survival assay was performed to determine the ability of the *B*. *pseudomallei* WT and SCV to replicate intracellularly. The assay was performed as previously described [20] with slight modification. Briefly, the A549 cells were infected with *B*. *pseudomallei* harvested at mid-logarithmic phase at a multiplicity of infection (MOI) of 1:10 and 1:100 for 2 hours. Following incubation at 37°C in a humidified 5% CO_2_, the infected monolayers were washed twice with sterile phosphate buffered saline (PBS). Fresh RPMI medium containing kanamycin (1 mg/ml) was added, followed by an additional 2 hours incubation to kill the extracellular bacteria. Thereafter, the cell monolayers were washed twice with sterile PBS and further incubated for 0, 2, 4, 6, 8, 10 and 12 hours in RPMI medium containing kanamycin (50 μg/ml). At each time point, the infected monolayers were then lysed using PBS containing 0.5% tergitol and 1% bovine serum albumin (BSA). Serial dilutions of the lysates were plated onto NA and the numbers of viable intracellular bacteria were determined [21]. The assay was performed in triplicate and the results were averaged. A non-invasive strain of *E*. *coli* was used as negative control.

### Cytotoxicity assay

A cytotoxicity assay that determines the level of lactate dehydrogenase (LDH) was performed using a CytoTox 96 non-radioactive kit (Promega, Madison, WI). Briefly, A549 cells were seeded into 96-well flat-bottom tissue culture plates and were incubated overnight until 80–90% of confluency is reached. The A549 cells monolayers were infected with *B*. *pseudomallei* WT and SCV at MOI of 1:10 and 1:100 for 0, 2, 4, 6, 8, 10 and 12 hours at 37°C in 5% CO_2_. Supernatants (50 μl) from each well were transferred into a new 96-well enzymatic assay plate. Substrate (50 μl) was then added to each well and incubated in the dark for 30 minutes period at room temperature. The reaction was stopped using 50 μl of stop solution and optical density measured at 490 nm. The infected monolayers LDH levels were normalized to maximum release of LDH (in which the A549 cells were lysed using Triton X-100 solution at a final concentration of 0.8% (v/v)). The experiment was performed in triplicate with LDH released from infected A549 cells designated as experimental LDH, the LDH released after total lysis of uninfected A549 cells designated as maximal LDH and the LDH released by uninfected A549 cells designated as target spontaneous LDH. Cytotoxicity was calculated as follows:

$$\%\;cytotoxicity\;=\;\frac{experimental\;LDH\;–\;target\;spontaneous\;LDH}{maximal\;LDH\;-\;target\;spontaneous\;LDH}$$

### Total protein extractions from *B*. *pseudomallei* exposed to A549 cells.

Approximately, 3.75 x 10^6^ A549 cells were seeded into T-75 tissue culture flask in 10 ml of RPMI 1640 (Sigma-Aldrich) supplemented with 10% FBS (Hyclone) and incubated overnight at 37°C with 5% CO_2_. The A549 cells were grown to 80–90% confluency and the monolayers were washed twice with PBS. The A549 cells were then exposed to *B*. *pseudomallei* at a MOI of 1:100 [18]. Following 2 hours incubation at 37°C, the supernatants were collected and centrifuged at 400 ×*g* at 4°C for 3 minutes to remove traces of the A549 cells. A loopfull of the supernatant from both variants (WT and SCV) were streaked onto the Ashdown agar to observe colony morphology changes as compared with the pre-exposed WT and SCV. The colony size, colour, translucency, circumference (smooth or irregular) were recorded [12]. The remaining supernatant was centrifuged again at 14000 ×*g* at 4°C for 15 minutes to harvest the bacteria. The resulting bacterial pellet was then washed three times with PBS and resuspended in one ml of cold lysis buffer containing 5 mM EDTA, 100 μl of protease inhibitors (Sigma) and 1 μl (5000 units) of benzonase (Sigma) [22, 23]. The bacterial cells were disrupted by sonication (mechanical procedure) for 3 minutes on ice with 22% amplitude at 1 second pulse intervals, followed by centrifugation at 14,000 ×*g* for 3 minutes at 4°C in order to obtain the total bacterial protein. The supernatant containing the proteins was collected and passed through a 0.2 μm filter and stored at −80°C [18]. All experiments were performed as triplicates of three independent experiments.

### Two-dimensional (2D) gel electrophoresis

Two-dimensional (2D) gel electrophoresis was performed as previously described [18]. Briefly, the protein samples were cleaned using the 2D clean-up kit (GE Healthcare, Uppsala, Sweden) and the concentration was determined using Bradford protein assay (BioRad, USA). The immobiline dry strips (nonlinear, pH 3–10, 13 cm) were rehydrated for 16 hours at 20°C with 550 μg total protein resuspended in rehydration buffer (7 M urea, 2 M thiourea, 2% CHAPS, 20 mM dithiothreitol (DTT), 0.5% pharmalyte and 0.002% bromophenol blue). The proteins were then focused at 500 V for 500 Vh, 1000 V for 1000 Vh and 8000 V for 12 500 Vh, using Ettan IPGphor 3 (GE Healthcare Bio-Sciences), as previously described [24]. The strips were then subjected to two consecutive equilibration of 15 minutes each in 10 ml of equilibration buffer (75 mM Tris-HCL, pH 8.8; 6 M urea, 29.3% glycerol; 2% SDS and 0.002% bromophenol blue) containing 1% DTT and 2.5% iodoacetamide (IAA), respectively. The strips were transferred to a 12.5% SDS-PAGE gel and the second dimension separation was performed using current/gel of 15 mA for 30 minutes and 30 mA for approximately 2 hours. The gels were then stained using hot Coomassie blue R-350 stain (GE Healthcare, Uppsala, Sweden), scanned using a GS-800 calibrated densitometer and the resulting images were analysed using the software PDQuest Basic version 8.1 algorithm (Biorad, USA). A minimum of three biological replicate gels were performed to increase the reproducibility.

### Mass spectrometry (MALDI-TOF/TOF) analysis

Following the image analysis, the protein spots of interest were excised from the Coomassie stained gels and the gel plugs were placed in 1.5 ml Eppendorf tubes and sent for trypsin digestion using Trypsin gold kit (Promega, USA) according to standard protocol [25]. Briefly, the excised gel plugs were trypsinised using 10 μL of trypsin digest solution (12.5 μg/mL trypsin and 25 mM ammonium bicarbonate) and incubated overnight at 37°C. The digested peptides were extracted twice for 20 minutes each using 10–20 mL ACN containing 1% TFA, depending on the size of the gel piece. MALDI-TOF/TOF analysis was performed using 5800 Proteomics Analyser (AB Sciex) at the Proteomics International Pty Ltd, Australia. Briefly, the peptide samples were reconstituted in 2 mL standard diluent ACN:water (30:70). The samples were diluted 1:10 with matrix solution (CHCA, 10 mg/mL) and spotted on a 384-well Opti-TOF stainless steel plate. The first run of standard TOF MS was used to analyse the samples. The second run of MS/MS was set in the system focused on the 15 most intensive peaks of the first MS (to exclude peaks known to be trypsin). In MS mode, each spot was fired 400 times with the laser using 2800 J intensity. However, in MS/MS mode, each spot was fired 2000 times with the laser using 3900 J intensity. A mass range of 400–4000 amu with a focus mass of 2100 amu was used.

### Protein identification

The fingerprinting map obtained from MALDI-TOF mass spectrometer was used for protein identification. The peptide mass data were submitted to MASCOT and analysed using Mascot sequence matching software 2.3 (Matrix Science, London, UK) with Ludwig NR Database. The parameters for search were set as follow: oxidation of methionine: variable modification, mass values: monoisotopic, protein mass: unrestricted, 1 missed cleavage per peptide was allowed, peptide mass tolerance: ± 0.4 Da and fragment mass tolerance: ± 0.4 Da. The generated data were blast searched against the non-redundant NCBI library for annotated proteins database of *B*. *pseudomallei* K96243 (reference strain) using the Basic Local Alignment Search Tool (BLAST) (http://www.ncbi.nlm.nih.gov/) for further analysis. SignalP v.3.0 (http://www.cbs.dtu.dk/services/SignalP/) was used to predict the presence of signal peptides of the proteins and PSORTb v.2.0 (http://www.psort.org/psortb2/index.html) was used to predict the cellular location of the identified proteins.

In our previous study, we have reported on the protein profiles associated with the morphotypic and phenotypic differences in *B*. *pseudomallei* WT and SCV grown in laboratory conditions (pre-exposure to A549 cells) [18]. Thus, in this study, we also performed a comparison between the WT post-exposure vs WT pre-exposure and SCV post-exposure vs SCV pre-exposure [18] to A549 cells.

## Results

### Morphology

**Fig 1:**
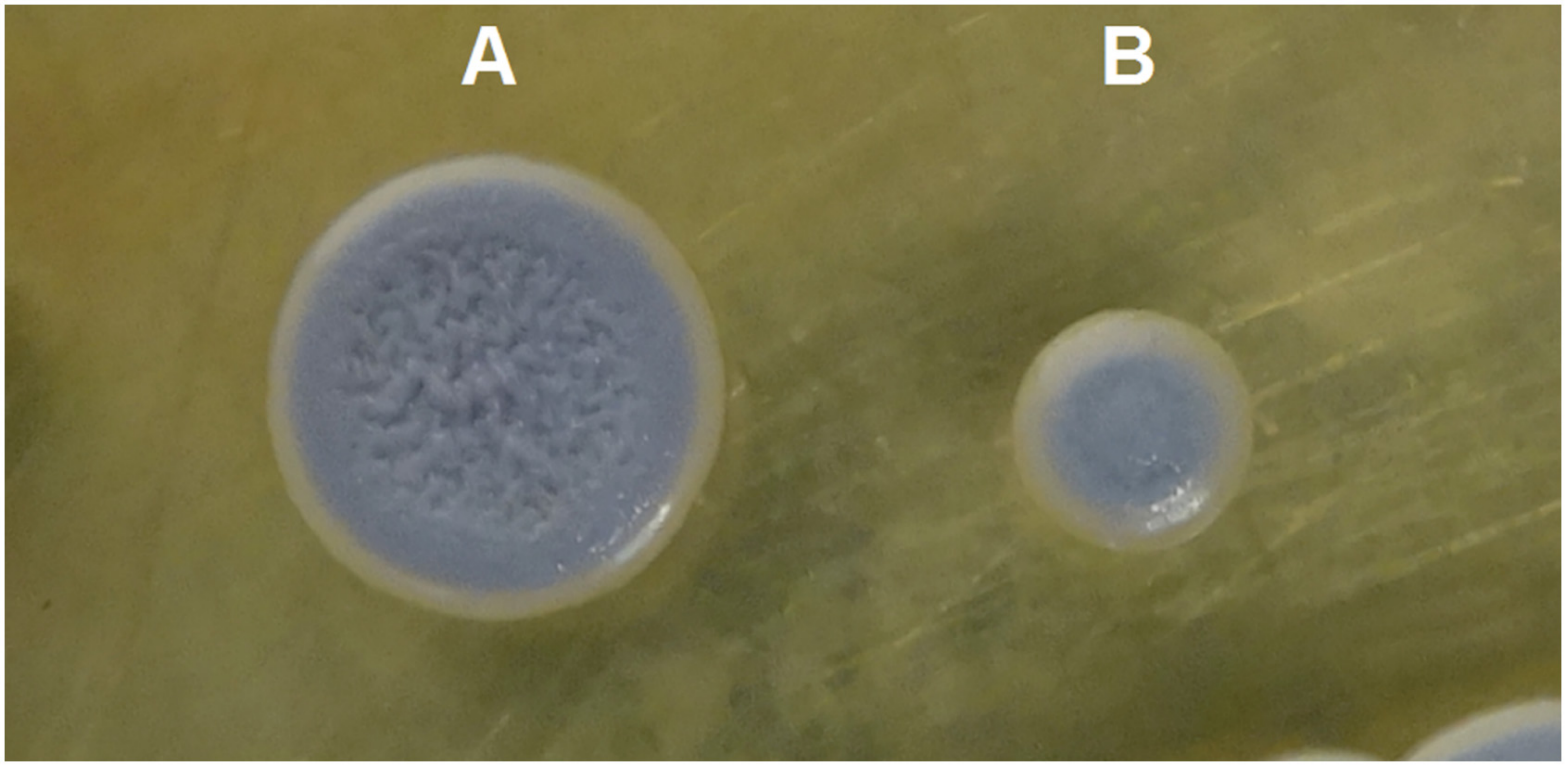
*Burkholderia pseudomallei* colony morphotypes. (A) Wild Type, WT and (B) small colony variant, SCV morphotypes grown for 4 days at 37°C on Ashdown agar.

The colony morphology of both the WT and SCV pre- and post-exposure to A549 cells for 4 days observed on Ashdown’s agar did not show any variation post-exposure to A549 cells (Fig 1). The WT was characterised by rough center, smooth in the outer edge, pale purple in colour and the diameter was more than 5 mm while the SCV showed smooth center and outer edge and less than 2 mm in diameter.

### Intracellular survival assay

**Fig 2:**
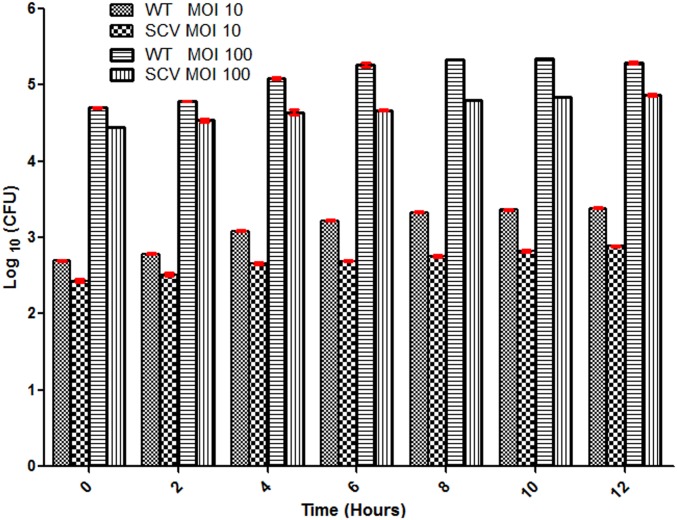
Intracellular survival of *Burkholderia pseudomallei* WT and SCV in A549 cells. Monolayers were infected with *B*. *pseudomallei* WT and SCV at a MOI of 1:10 and 1:100 and the intracellular loads of bacteria were quantified over 12 hours post-infection. The values are the means of each of three independent experiments, each performed in triplicate and error bar represents the standard deviation.

Another possible factor that might be associated with the virulence of *B*. *pseudomallei* WT and SCV colony variants is their ability to survive and effectively replicate intracellularly in A549 cells, and this was assessed over a 12 hour-period as previously described [26] (Fig 2). At the MOI of 1:10, significant differences were observed in the intracellular survival among both variants at different time-points post-infection. In comparison with the SCV, the WT variant demonstrated a marked and gradual increase in the number of intracellular bacteria from zero to 12 hours post-infection. The results showed that the numbers of intracellular bacteria of the WT variant [log_10_ colony forming unit (cfu) value of 2.70 and 3.39 at zero hour and 12 hour post-infection, respectively] was significantly higher than SCV (log_10_ cfu value of 2.44 and 2.89 at zero hour and 12 hours post-infection, respectively). Similarly, at the MOI of 1:100, the number of intracellular WT variant showed a noticeable and gradual increase compared with the SCV. The numbers of intracellular WT (log_10_ cfu value of 4.71 and 5.35 at zero hour and 10 hours post-infection, respectively) was significantly higher than the SCV (log_10_ cfu value of 4.44 and 4.85 at zero hour and 10 hours post-infection, respectively). However, in contrast to the MOI 1:10, after 10 hours post-infection, the intracellular survival and replication in the WT decreased (log_10_ cfu values of 5.30), whereas the SCV continuously demonstrated gradual increase (log_10_ cfu values of 4.87) in its intracellular survival and replication at the MOI 1:100.

### Cytotoxicity assay

**Fig 3:**
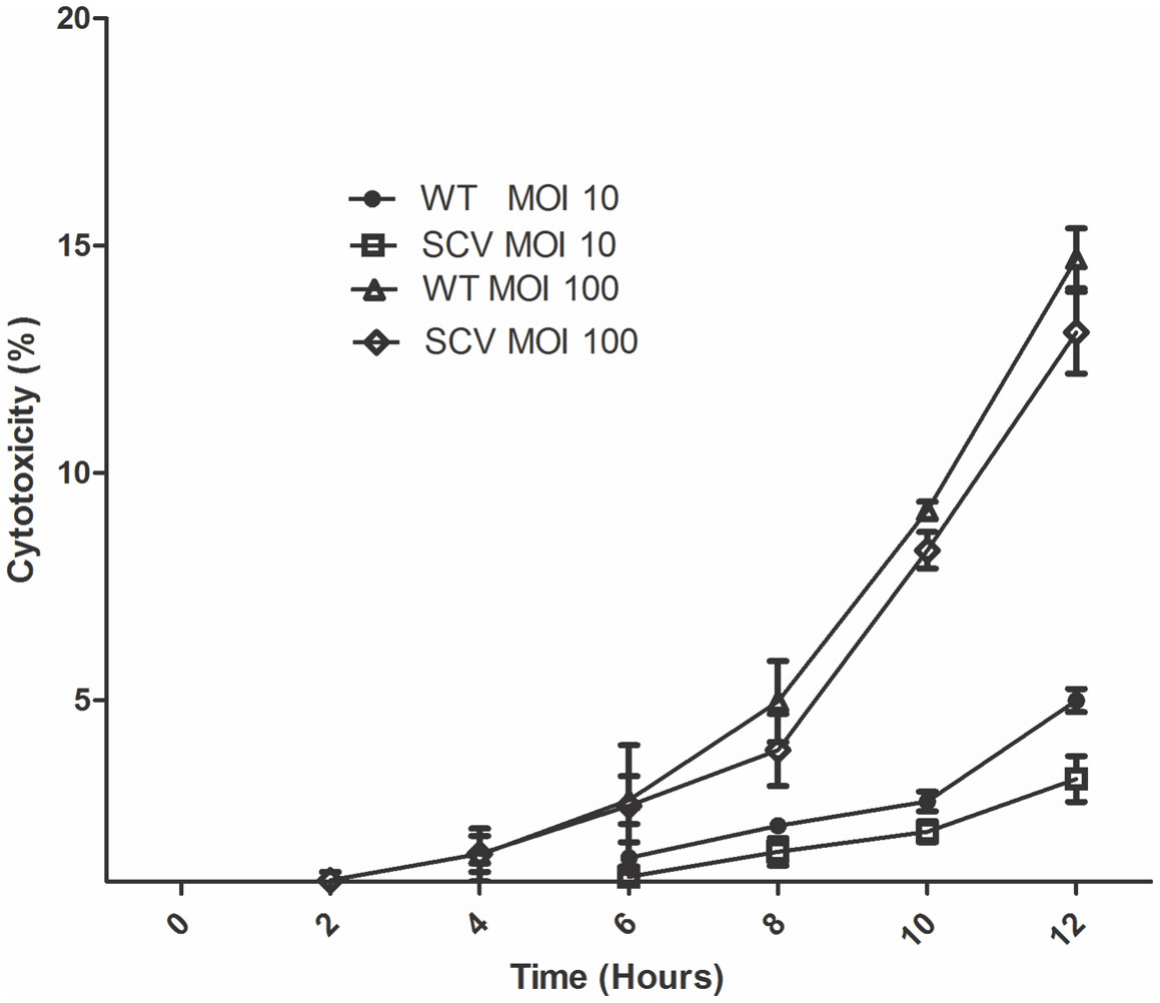
Percentage of cytotoxicity to A549 epithelial cells. Cells were infected with the bacteria at a MOI 1:10 and 1:100 for 0, 2, 4, 6, 8, 10, and 12 hours. The values are the means ± the standard deviation of each of three independent experiments, each performed in triplicate.

Exposure of A549 cells to *B*. *pseudomallei* WT and SCV grown to mid-logarithmic phase at MOI 1:10 and 1:100 demonstrated a mild increase in the percentage of cytotoxicity to A549 cells over 12 hours post-infection (Fig 3). The cytotoxicity to A549 was less than 5% at MOI 1:10, at zero to 12 hours post-infection and less than 15% at MOI 1:100. No significant differences were found in the percentage of cytotoxicity induced by both the WT and SCV morphotypes at both MOIs. At MOI 1:10, the percentages of cytotoxicity of WT were 0.21±0.14 and 4.99±0.44 at zero and 12 hours post-infection, respectively and 0.15±0.11 and 3.26± 0.87, respectively for the SCV. At MOI 1:100, the results demonstrated that the percentages of cytotoxicity of WT post-infection were 0.45±0.39 and 9.17±0.34 at zero to 10 hours; however, in SCV were 0.44±0.20 and 8.3±0.70, respectively. In addition, cytotoxicity assays demonstrated that WT caused the A549 cells to release more LDH than SCV at 12 hours post-infection at MOI 1:100, in which cytotoxicity percentage for WT was 14.72± 1.14 and for SCV was 13.09± 1.56, however, the difference was not significant.

### Profiling and identification of *B*. *pseudomallei* proteins

#### WT vs SCV (post-exposure to A549 cells)

**Fig 4:**
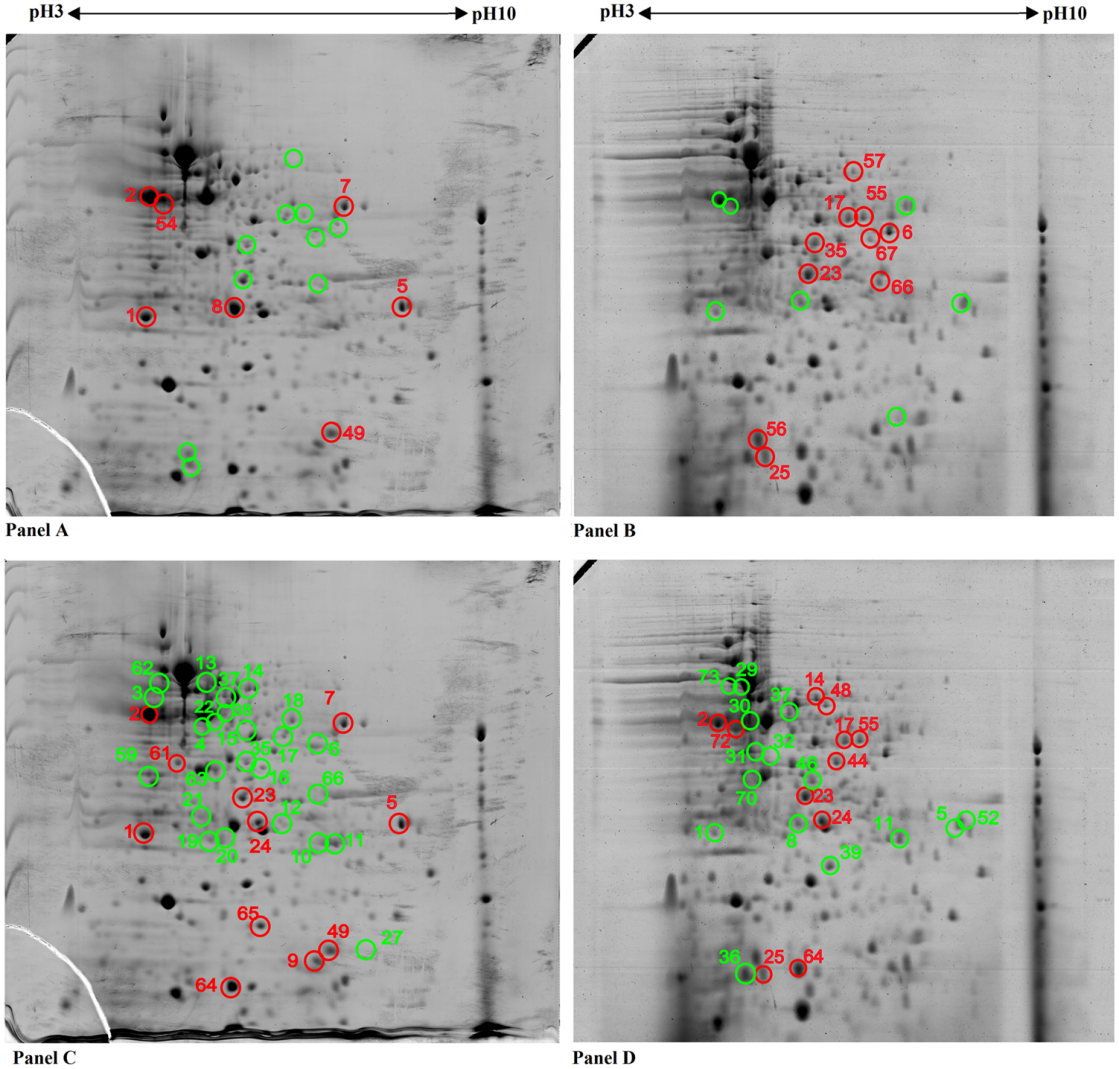
Proteomic profiles of *Burkholderia pseudomallei*. Regulation of protein spots of SCV post-exposure (Panel B) in comparison with WT post-exposure (Panel A). WT post-exposure gel (Panel C) showing regulation of protein spots in comparison to WT pre-exposure (18) and SCV post-exposure gel (Panel D) showing regulation of protein spots in comparison to SCV pre-exposure (18). In the post-exposure, the bacteria was exposed to A549 cells for 2 hours, then the recovered bacteria were subjected to sonication to lyse the bacteria followed by proteins extraction to be used in 2-D-GE. A total of 550 mg of proteins was separated on an IPG strip pH 3–10 in the first dimension, followed by the separation on SDS-12% PAGE for the second-dimension separation. Coomassie blue R-350 stain was used to visualize the separated proteins. The up-regulated protein spots are indicated by red circles and down-regulated spots indicated by green circles. Protein spot numbers relate to information provided in the text and Tables 1–3.

Protein expression was compared between *B*. *pseudomallei* strain WT versus SCV post-exposure to A549 cells. Proteomic analysis of *B*. *pseudomallei* WT post-exposure uncovered about 260 protein spots versus 259 protein spots for SCV post-exposure to A549 cells (Fig 4). Of these 19 protein spots were found to be differentially expressed in the SCV post-exposure (12 up-regulated and 7 down-regulated) compared with the WT. Among these 19 differentially expressed proteins, 17 were identified (10 up-regulated and 7 down-regulated) with confidence using MS and database search (Table 1). The remaining two up-regulated protein spots were of low quantity and in insufficient quantities for the MALDI-TOF/TOF analysis.

**Table 1:** Differentially expressed protein of *Burkholderia pseudomallei* WT vs SCV (post-exposure to A549 cells) identified using MALDI-TOF/TOF analysis.

Spot No. ^a^	Protein Name/Function	Locus Taq	Sequence Coverage (%)	Peptides Matched	Exp/Theo (MW)	Exp/Theo (*pI*)	Score	SignalP^b^	PSORTb ^c^	Fold change	*P*-value
**UP-REGULATED PROTEINS**											
**Metabolism**											
**A. Energy Production and Conversion**											
35	Malate dehydrogenase (Mdh)	BPSS1722	31	10	34.92/34.88	5.70/5.70	435	-	Multiple sites	2.90	0.001
57	Betaine aldehyde dehydrogenase (BetB)	BPSS1354	15	5	52.14/52.1	5.41/5.41	246	-	Cytoplasmic	NA	NA
66	Succinyl-CoA ligase [ADP-forming] subunit alpha (SucD)	BPSL0780	19	5	32.72/30.64	8.81/6.25	175	-	Cytoplasmic	2.71	0.01
25	Putative uncharacterised protein	BPSL2288	10	2	14.36/14.15	5.33/5.34	71	-	Cytoplasmic	3.47	0.008
**B. Amino acid Transport and Metabolism**											
55	Ornithine carbamoyltransferase (ArcB)	BPSL1744	10	2	37.99/37.89	6.1/6.17	141	-	Cytoplasmic	2.94	0.01
23	2,3,4,5 tetrahydropyridine -2,6-carboxylate N-succinyltransferase (DapD)	BPSL2169	19	5	29.52/29.48	5.68/5.66	371	-	Cytoplasmic	2.03	0.05
**C. Carbohydrate Transport and Metabolism**											
17	Fructose-bisphosphate aldolase (CbbA)	BPSL0798	17	6	38.49/38.31	5.97/5.87	361	-	Cytoplasmic	2.70	0.006
6	Glyceraldehyde 3-phosphate dehydrogenase 1 (GapA)	BPSL2952	40	12	36.08/36.12	6.37/6.37	1055	-	Cytoplasmic	2.37	0.01
**D. More than one function**											
**1. Amino acid Transport and Metabolism and Nucleotide Transport and Metabolism**											
67	Ribose-phosphate pyrophosphokinase (Prs)	BPSL0521	8	2	34.15/34.08	6.01/6.01	71	-	Cytoplasmic	2.62	0.01
**Cellular Processes**											
**A. Signal Transduction Mechanisms**											
56	DksA/traR C4-type zinc finger family protein	BPSL0205	28	3	16.00/15.88	5.26/5.24	91	-	Cytoplasmic	2.04	0.04
**DOWN-REGULATED PROTEINS**											
**Metabolism**											
**A. Lipid Metabolism**											
1	succinyl-CoA:3-ketoacid-coenzyme A transferase subunit B (ScoB)	BPSL1954	44	12	22.27/22.29	4.70/4.70	818	-	Cytoplasmic	-6.75	0.02
8	Succinyl-CoA:3-ketoacid-coenzyme A transferase subunit A (ScoA)	BPSL1955	43	9	25.23/25.09	5.56/5.55	874	-	Cytoplasmic	-10.23	0.0007
7	Acetyl-CoA acetyltransferase (PhbA)	BPSL1535	32	10	40.49/40.41	6.62/6.62	973	-	Cytoplasmic	-2.28	0.02
**B. Amino acid Metabolism**											
5	Lysine-arginine-ornithine transport system, binding exported protein (ArgT)	BPSS0269	53	13	28.32/28.33	8.61/8.61	1127	+	Periplasmic	-2.59	0.001
**C. Carbohydrate Transport and Metabolism**											
2	Enolase: phosphopyruvate hydratase (Eno)	BPSL2270	31	13	45.65/45.55	4.81/4.81	1187	-	Cytoplasmic	-2.13	0.04
**Cellular Processes**											
**A. Cell cycle control, Cell Division, Chromosome Partitioning**											
54	Cell division protein (ftsZ)	BPSL3020	29	12	41.57/41.47	4.87/4.86	400	-	Cytoplasmic	NA	0.004
**Poorly characterised**											
49	Putative exported protein	BPSL2413	30	5	15.842/15.663	6.9/6.91	92	+	Multiple sites	NA	0.01

^a^Protein spot corresponding to position on gel (Fig 4 Panels A and B),

^b^Output of computer algorithms that predict the presence (1) or absence (-) of signal peptide,

^c^Output of computer algorithms that predict the subcellular location of protein. Of the up-regulated proteins, the PSORT algorithm predicted 90% to be cytoplasmic and 10% with multiple localization sites. Amongst the down-regulated proteins, 71.4% were of cytoplasmic, 14.3% of periplasmic and 14.3% were of multiple localization sites. NA: Protein spot not available.

#### WT (pre-exposure vs post-exposure to A549 cells)

In comparison of protein expression between WT pre-exposure [18] and WT post- exposure to A549 cells (Fig 4; WT post-exposure (Panel C)), 36 protein spots were observed to be differentially expressed in WT post-exposure (11 up-regulated and 25 down-regulated). Of these, 35 were subsequently identified using MS and database search. The remaining one protein spot (1 down-regulated protein) could not be identified by MALDI-TOF/TOF analysis (Table 2). Among the down-regulated proteins in WT post-exposure, 24 were identified and one of these *i*.*e*. GroEL1 (n = 8) was present as isoforms (Table 3).

**Table 2:** Differentially expressed protein of *Burkholderia pseudomallei* WT (pre-exposure vs post-exposure (18) to A549 cells) identified using MALDI-TOF/TOF analysis.

Spot No. ^a^	Protein Name/Function	Locus Taq	Sequence Coverage (%)	Peptides Matched	Exp/Theo (MW)	Exp/Theo (*pI*)	Score	SignalP^b^	PSORTb ^c^	Fold change	*P*-value
**UP-REGULATED PROTEINS**											
**Metabolism**											
**A. Lipid Metabolism**											
1	succinyl-CoA:3-ketoacid-coenzyme A transferase subunit B (ScoB)	BPSL1954	44	12	22.27/22.29	4.70/4.70	818	-	Cytoplasmic	3.83	0.001
7	Acetyl-CoA acetyltransferase (PhbA)	BPSL1535	32	10	40.49/40.41	6.62/6.62	973	-	Cytoplasmic	3.56	0.01
**B. Energy Production and Conversion**											
24	Ferredoxin—NADP reductase (Fpr)	BPSL0241	42	10	30.42/28.69	5.65/5.78	380	-	Cytoplasmic	3.55	0.000
**C. Amino acid Transport and Metabolism**											
5	Lysine-arginine-ornithine transport system, binding exported protein (ArgT)	BPSS0269	53	13	28.32/28.33	8.61/8.61	1127	+	Periplasmic	3.56	0.01
23	2,3,4,5-tetrahydropyridine-2,6-carboxylate N-succinyltransferase (DapD)	BPSL2169	19	5	29.52/29.38	5.68/5.66	371	-	Cytoplasmic	3.13	0.0007
**D. Carbohydrate Transport and Metabolism**											
2	Enolase: phosphopyruvate hydratase (Eno)	BPSL2270	31	13	45.65/45.55	4.81/4.81	1187	-	Cytoplasmic	2.58	0.02
**E. Nucleotide Transport and Metabolism**											
64	Nucleoside diphosphate kinase (Ndk)	BPSL1510	26	2	15.55/15.43	5.61/5.61	83	-	Extracellular	2.07	0.001
**F. Inorganic ion transport and metabolism**											
9	Putative ferritin DPS-family DNA binding protein (Dps)	BPSL2863	67	11	18.13/18.02	5.95/5.95	927	-	Cytoplasmic	16.58	0.000
**Cellular Processes**											
**A. Posttranslational Modification, Protein Turnover, Chaperones**											
65	Peptidyl-prolyl cis-trans isomerase B (PpiB)	BPSL2246	30	5	17.87/17.76	5.94/5.94	240	-	Cytoplasmic	2.82	0.02
**B. Cell cycle control, cell division, chromosome partitioning**											
61	Translocator protein (BipD)	BPSS1529	30	7	34.01/33.98	5.06/5.13	336	-	Extracellular	NA	NA
**Poorly characterised**											
49	Putative exported protein	BPSL2413	30	5	15.84/15.66	6.9/6.91	92	+	Multiple sites	3.27	0.03
**DOWN-REGULATED PROTEINS**											
**Metabolism**											
**A. Energy Production and Conversion**											
35	Malate dehydrogenase (Mdh)	BPSS1722	31	10	34.92/34.88	5.70/5.70	435	-	Multiple sites	-3.71	0.0002
66	Succinyl-CoA ligase [ADP-forming] subunit alpha (SucD)	BPSL0780	19	5	32.72/30.64	8.81/6.25	175	-	Cytoplasmic	-6.26	0.005
**B. Amino acid Transport and Metabolism**											
18	Succinylornithine transaminase (ArgM)	BPSL2390	15	4	43.69/43.59	6.03/6.03	279	-	Cytoplasmic	-2.11	0.01
**C. Carbohydrate Transport and Metabolism**											
6	Glyceraldehyde 3-phosphate dehydrogenase 1(GapA)	BPSL2952	40	12	36.08/36.12	6.37/6.37	1055	-	Cytoplasmic	-3.82	0.003
17	Fructose-bisphosphate aldolase (CbbA)	BPSL0798	17	6	38.49/38.31	5.97/5.87	361	-	Cytoplasmic	-2.21	0.003
**D. Coenzyme Metabolism**											
14	Adenosylhomocysteinase (AhcY)	BPSL3290	24	9	52.17/52.07	5.73/5.73	500	-	Cytoplasmic	-3.70	0.00113
**E. Secondary Metabolites Biosynthesis, Transport and Catabolism**											
16	Putative non-ribosomally encoded peptide/polyketide synthase (PhyH)	BPSS1183	29	8	35.38/35.28	5.77/5.77	376	-	Cytoplasmic	-4.43	0.003
**F. More than one function**											
**1. Amino acid Transport and Metabolism and Coenzyme Transport and Metabolism**											
22	Putative ketol-acid reductoisomerase	BPSS0305	10	4	37.92/37.79	5.31/5.31	117	-	Cytoplasmic	NA	NA
**2. Secondary metabolites biosynthesis, transport and catabolism and Lipid transport and metabolism**											
10	Acetoacetyl-CoA reductase (PhbB)	BPSS1916	43	13	26.44/26.29	6.60/6.30	1075	-	Cytoplasmic	-4.88	0.00001
11	Putative dehydrogenase	BPSL1167	19	4	26.27 /26.57	6.32/6.31	371	-	Cytoplasmic	-5.02	0.001
**Cellular Processes**											
**A. Signal Transduction Mechanisms**											
63	Universal stress family protein	BPSS1140	36	10	33.85 /34.33	5.46/5.47	394	-	Cytoplasmic	NA	NA
**B. Posttranslational Modification, Protein Turnover, Chaperones**											
20	Protein-L-isoaspartate O-methyltransferase (Pcm)	BPSL2648	40	7	24.01/24.02	5.55/5.55	374	-	Cytoplasmic	-2.48	0.00009
3	60 kDa chaperonin 1 (GroEL1)	BPSL2697	30	13	57.08/57.12	5.13/5.13	1234	-	Cytoplasmic	NA	NA
4	60 kDa chaperonin 1 (GroEL1)	BPSL2697	26	13	57.08/57.12	5.13/5.16	1140	-	Cytoplasmic	-5.63	0.0003
12	60 kDa chaperonin 1 (GroEL1)	BPSL2697	25	9	57.49/57.12	5.13/5.13	495	-	Cytoplasmic	-19.86	0.0001
13	60 kDa chaperonin 1 (GroEL1)	BPSL2697	29	13	57.08/57.11	5.13/5.34	674	-	Cytoplasmic	NA	NA
21	60 kDa chaperonin 1 (GroEL1)	BPSL2697	27	13	19.97/57.11	5.4/5.13	180	-	Cytoplasmic	NA	NA
37	60 kDa chaperonin 1 (GroEL1)	BPSL2697	31	14	57.49/57.12	5.13/5.13	793	-	Cytoplasmic	-5.78	0.001
59	60 kDa chaperonin 1 (GroEL1)	BPSL2697	12%	5	57.08 /57.12	5.13/5.13	242	-	Cytoplasmic	-2.81	0.02
62	60 kDa chaperonin 1 (GroEL1)	BPSL2697	25	9	57.08/ 57.12	5.13/5.13	441	-	Cytoplasmic	NA	NA
**Information Storage and Processing**											
**A. Transcription**											
15	DNA-directed RNA polymerase subunit alpha (RpoA)	BPSL3187	28	7	35.66/35.55	5.76/5.76	261	-	Cytoplasmic	NA	NA
**B. Translation, Ribosomal Structure, Biogenesis**											
27	Putative PTS system, EIIa component	BPSL0532	27	3	13.67/13.53	6.49/6.49	152	-	Cytoplasmic	-2.98	0.009
38	Elongation factor Tu (EF-Tu)	BPSL3215	7	3	42.91/42.86	5.36/5.34	138	-	Cytoplasmic	NA	NA
**General Function Prediction Only**											
19	Putative uncharacterised protein	BPSS1924	7	2	23.15/23.04	5.33/5.31	83	-	Cytoplasmic	-3.21	0.0003

^a^Protein spot corresponding to position on gel (Fig 4 Panel C),

^b^Output of computer algorithms that predict the presence (1) or absence (-) of signal peptide,

^c^Output of computer algorithms that predict the subcellular location of protein. Of the up-regulated proteins, the PSORT algorithm predicted that 63.6% were of cytoplasmic, 9.1% of periplasmic, 18.2% of extracellular and 9.1% of multiple localization sites. Amongst the down-regulated proteins, 94.1% were of cytoplasmic in origin and 5.9% were of multiple localization sites. NA: Protein spot not available.

**Table 3:** Identical protein spots of *Burkholderia pseudomallei* colony morphovariants among different conditions.

Protein Name	Spot Number	Condition
**60 kDa chaperonin 1 [GroEL1]**	3, 4, 12, 13, 21, 37, 59, 62	WT (pre-exposure vs post-exposure to A549 cells) [18]
**60 kDa chaperonin 1 [GroEL1]**	29, 30, 37, 70, 73	SCV (pre-exposure vs post-exposure to A549 cells) [18]
**2-oxoisovalerate dehydrogenase beta subunit (BkdA2)**	31, 32	SCV (pre-exposure vs post-exposure to A549 cells) [18]

#### SCV (pre-exposure vs post-exposure to A549 cells)

Similarly, comparison of protein expression between SCV pre-exposure [18] and SCV post- exposure to A549 cells was performed (Fig 4; SCV post-exposure (Panel D)), only 27 protein spots were observed to be differential expressed (11 up-regulated and 16 down-regulated) in SCV post-exposure when compared with SCV pre-exposure to A549 cells. All up-regulated proteins were identified using MS and database search whereas one protein spot among the down-regulated proteins could not be identified by the MALDI-TOF/TOF analysis (Table 4) and two of these *i*.*e*. BkdA2 (n = 2) and GroEL1 (n = 5) were present as isoforms (Table 3).

**Table 4:** Differentially expressed protein of *Burkholderia pseudomallei* SCV (pre-exposure vs post-exposure (18) to A549 cells) identified using MALDI-TOF/TOF analysis.

Spot No. ^a^	Protein Name/Function	Locus Taq	Sequence Coverage (%)	Peptides Matched	Exp/Theo (MW)	Exp/Theo (*pI*)	Score	SignalP^b^	PSORTb ^c^	Fold change	*P*-value
**UP-REGULATED PROTEINS**											
**Metabolism**											
**A. Energy Production and Conversion**											
24	Ferredoxin-NADP reductase (Fpr)	BPSL0241	42	10	30.42/28.69	5.65/5.78	380	-	Cytoplasmic	5.13	0.009
25	Putative uncharacterised protein	BPSL2288	10	2	14.36 /14.15	5.33/5.34	71	-	Cytoplasmic	9.65	0.001
44	Flavohemoprotein (HmpA)	BPSL2840	11	3	43.49/43.42	6.1/6.09	87	-	Cytoplasmic	3.92	0.001
**B. Amino acid Transport and Metabolism**											
23	2,3,4,5-tetrahydropyridine-2,6-carboxylate N-succinyltransferase (DapD)	BPSL2169	19	5	29.52/29.38	5.68/5.66	371	-	Cytoplasmic	2.36	0.01
55	Ornithine carbamoyltransferase, catabolic (ArcB)	BPSL1744	10	2	37.99/37.89	6.10/6.17	141	-	Cytoplasmic	3.46	0.01
**C. Carbohydrate Transport and Metabolism**											
2	Enolase: phosphopyruvate hydratase (Eno)	BPSL2270	31	13	45.65/45.55	4.81/4.81	1187	-	Cytoplasmic	2.90	0.01
17	Fructose-bisphosphate aldolase (CbbA)	BPSL0798	17	6	38.49/38.31	5.97/5.87	361	-	Cytoplasmic	3.20	0.02
**D. Coenzyme Metabolism**											
14	Adenosylhomocysteinase (AhcY)	BPSL3290	24	9	52.17/52.07	5.73/5.73	500	-	Cytoplasmic	3.01	0.01
48	Phenylacetate-coenzyme A ligase (PaaA)	BPSL3045	21	6	47.74 /47.51	5.80/5.80	289	-	Cytoplasmic	2.56	0.04
**E. Nucleotide Transport and Metabolism**											
64	Nucleoside diphosphate kinase (Ndk)	BPSL1510	26	2	15.55/15.43	5.61/5.61	83	-	Extracellular	2.55	0.02
**Cellular Processes**											
**A. Cell motility and secretion**											
72	Flagellin (FliC)	BPSL3319	8	4	39.75/39.13	4.89/5.05	151	-	Cytoplasmic	2.14	0.008
**DOWN-REGULATED PROTEINS**											
**Metabolism**											
**A. Lipid metabolism**											
1	Succinyl-CoA:3-ketoacid-coenzyme A transferase subunit B (ScoB)	BPSL1954	44	13	22.27/22.29	4.70/4.70	818	-	Cytoplasmic	-2.77	0.02
8	Succinyl-CoA:3-ketoacid-coenzyme A transferase subunit A (ScoA)	BPSL1955	43	9	25.23/25.09	5.56/5.55	874	-	Cytoplasmic	-2.92	0.009
46	Acetyl-CoA acetyltransferase (PhbA)	BPSL1535	21	7	40.49/ 40.41	6.62/6.62	382	-	Cytoplasmic	-3.65	0.0006
**B. Energy Production and Conversion**											
31	2-oxoisovalerate dehydrogenase beta subunit (BkdA2)	BPSS2272	25	5	37.77/37.66	5.14/5.14	310	-	Cytoplasmic	-5.11	0.01
32	2-oxoisovalerate dehydrogenase beta subunit (BkdA2)	BPSS2272	25	5	37.77/37.66	5.14/5.14	310	-	Cytoplasmic	-5.28	0.01
**C. Amino acid Transport and Metabolism**											
5	Lysine-arginine-ornithine transport system, binding exported protein (ArgT)	BPSS0269	53	13	28.32/28.33	8.61/8.61	1127	+	Periplasmic	-6.62	0.04
**D. Nucleotide Mransport and Metabolism**											
52	Adenylate kinase (Adk)	BPSL0875	27	6	24.16/24.04	7.74/7.74	282	-	Cytoplasmic	-4.26	0.01
**E. More than one function**											
**1. Secondary Metabolites Biosynthesis, Transport and Catabolism and Lipid Transport and Metabolism**											
11	Putative dehydrogenase	BPSL1167	19	4	26.27 /26.57	6.32/6.31	371	-	Cytoplasmic	-2.93	0.01
**Cellular Processes**											
**A. Posttranslational Modification, Protein turnover, Chaperones**											
36	HSP20/alpha crystallin family protein	BPSS2288	32	4	15.72/15.89	5.14/5.14	247	-	Multiple sites	-2.35	0.0134
29	60 kDa chaperonin 1 (GroEL1)	BPSL2697	29	11	57.08/57.12	5.13/5.13	630	-	Cytoplasmic	-4.08	0.007
30	60 kDa chaperonin 1 (GroEL1)	BPSL2697	24	9	57.49/57.12	5.13/5.13	683	-	Cytoplasmic	-2.26	0.0004
37	60 kDa chaperonin 1 (GroEL1)	BPSL2697	31	14	57.49/57.12	5.13/5.13	793	-	Cytoplasmic	-2.16	0.04
70	60 kDa chaperonin 1 (GroEL1)	BPSL2697	13	5	57.08/57.12	5.13/5.05	281	-	Cytoplasmic	NA	NA
73	60 kDa chaperonin 1 (GroEL1)	BPSL2697	29	3	57.08/57.12	5.13/5.13	147	-	Cytoplasmic	-4.74	0.04
39	Putative oxidoreductase	BPSL2748	38	8	23.84/23.73	5.75/5.75	327	-	Cytoplasmic	-3.95	0.0007

^a^Protein spot corresponding to position on gel (Fig 4 Panel D),

^b^Output of computer algorithms that predict the presence (1) or absence (-) of signal peptide,

^c^Output of computer algorithms that predict the subcellular location of protein. Of the up-regulated proteins, the PSORT algorithm predicted that 90.9% were of cytoplasmic and 9.1% of extracellular origin. Amongst the down-regulated proteins, 80% were of cytoplasmic in origin, 10% of periplasmic and 10% were of multiple localization sites. NA: Protein spot not available.

### *In silico* analysis

#### WT vs SCV (post-exposure to A549 cells)

**Fig 5:**
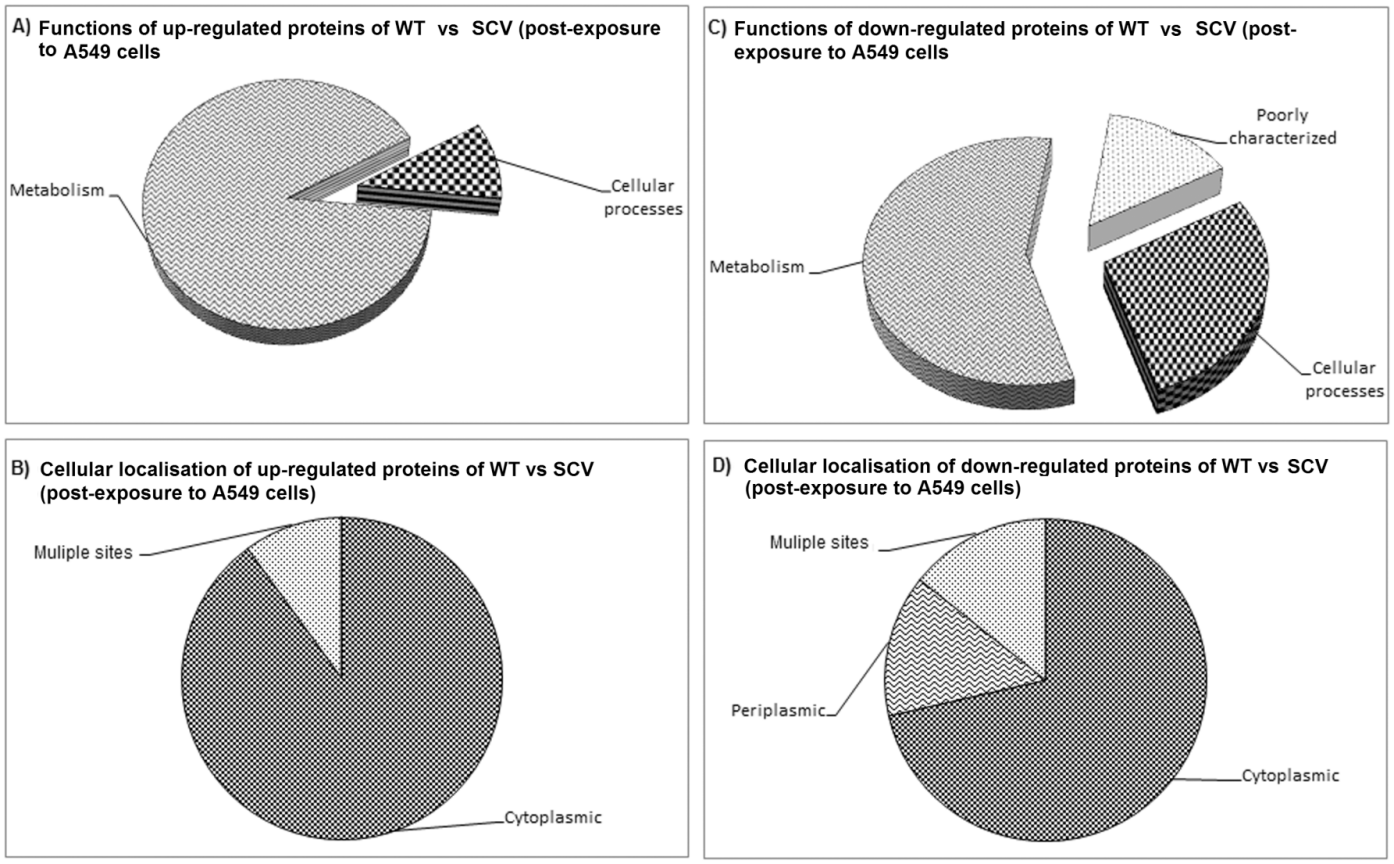
Protein categories and the subcellular localisation of *Burkholderia pseudomallei* WT vs SCV (post-exposure to A549 cells). Functional protein categories were predicted using COG, while subcellular localisation was predicted using PSORT. Panels A and B refer to functions and cellular location of the up-regulated proteins, respectively. Panels C and D refer to functions and cellular location of the down-regulated proteins, respectively.

*In silico* approaches were utilised to identify the functional categories and subcellular localisation of the differentially expressed proteins. Among the 10 up-regulated proteins detected in the SCV comparing with WT post-exposure to A549 cells, 9 proteins (Mdh, BetB, SucD, BPSL2288, CbbA, GapA, ArcB, DapD, and Prs) were involved in metabolism and one (BPSL0205) in cellular processes (Table 1; Fig 5A). However, using the PSORT algorithm to predict the subcellular localisation revealed that 90% of the proteins were of cytoplasmic and 10% were of multiple localisation sites (Fig 5B). On the other hand, among the 7 down-regulated proteins, five (ScoB, ScoA, PhbA, ArgT and Eno) were involved in metabolism, one in cellular processes (FtsZ) and one (BPSL2413) was poorly characterised with unknown function prediction (Fig 5C). Of the 7 down-regulated proteins, 71.4% were of cytoplasmic origin, 14.3% of periplasmic and 14.3% were of multiple localisation sites (Fig 5D). ArgT and BPSL2413 were the only proteins predicted to contain signal sequence cleavage sites secreted via the classical sec-pathway using the SignalP version 4.0.

#### WT (pre-exposure vs post-exposure to A549 cells)

**Fig 6:**
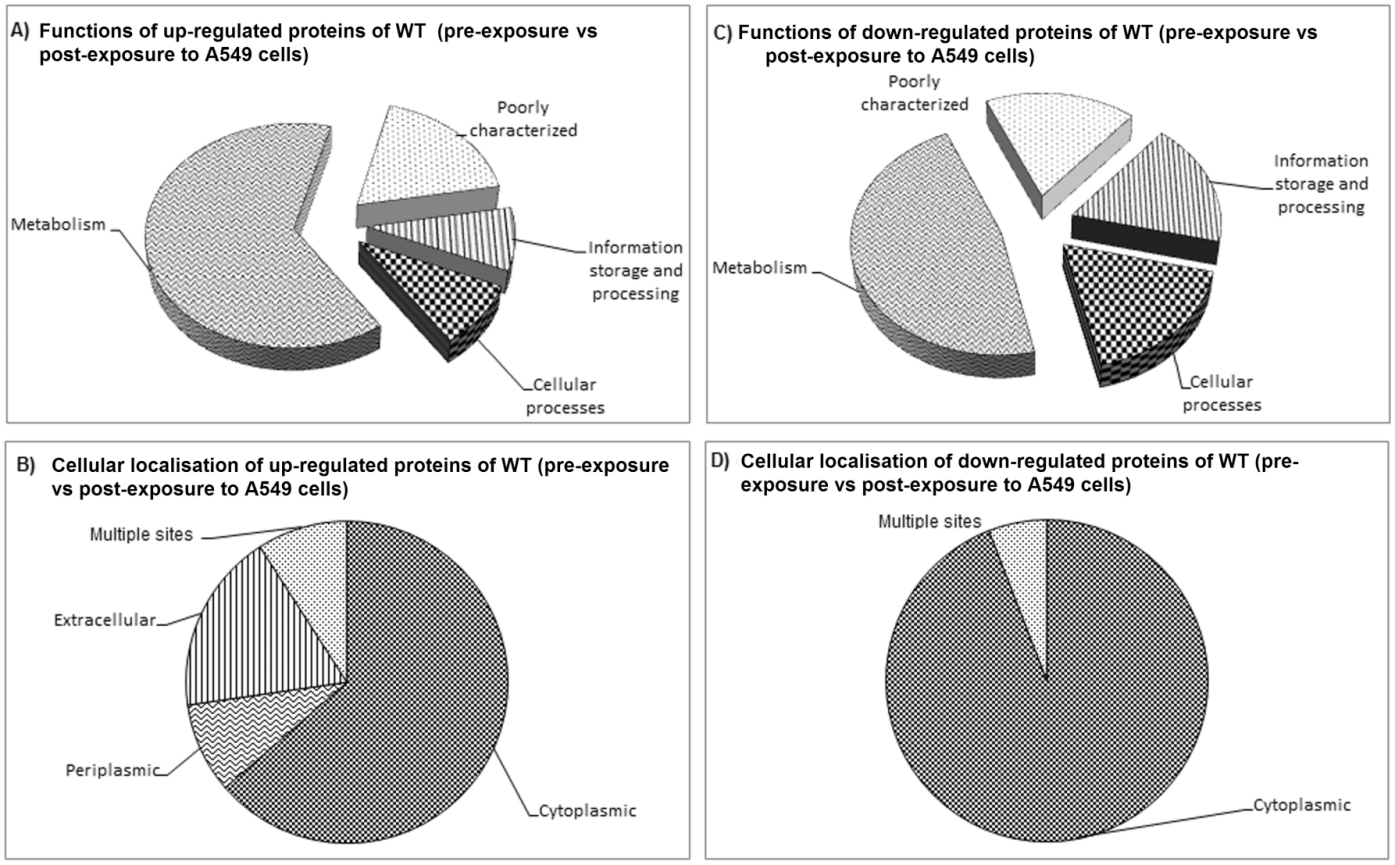
Protein categories and the subcellular localisation of *Burkholderia pseudomallei* WT (pre-exposure vs post-exposure to A549 cells). Functional protein categories were predicted using COG, while subcellular localisation was predicted using PSORT. Panels A and B refer to functions and cellular location of the up-regulated proteins, respectively. Panels C and D refer to functions and cellular location of the down-regulated proteins, respectively.

Amongst the 11 up-regulated proteins detected in the WT post-exposure comparing with WT pre-exposure to A549 cells, eight (ArgT, DapD, ScoB, PhbA, Eno, Fpr, Ndk and DPS) were involved in bacterial metabolism, two (PpiB and BipD) in cellular processes and one (BPSL2413) was of unknown function (Table 2; Fig 6A). However, localisation prediction revealed that, of the 11 up-regulated proteins, 63.6% were of cytoplasmic, 9.1% of periplasmic, 18.2% of extracellular and (9.1%) of multiple localisation sites (Fig 6B). On the other hand, among the 17 down-regulated proteins, ten (GapA, CbbA, Mdh, SucD, PhbB, PhyH, ArgM, BPSL1167, BPSS0305, and AhcY) were involved in metabolism, three (BPSS1140, PCM, and GroEL1 (8 spots)) in cellular processes (Fig 6C), two (RpoA and BPSL0532) in information storage and processing, one (EF-Tu) in information storage, processing and metabolism and one (BPSS1924) in general function prediction only. Of the 17 down-regulated proteins, most (94.1%) were of cytoplasmic in origin, and 5.9% were of multiple localisation sites (Fig 6D). Proteins that were predicted (using the SignalP version 4.0) to be secreted via the classical secretion pathway due to containing signal sequence cleavage sites includes ArgT and BPSL2413.

#### SCV (pre-exposure vs post-exposure to A549 cells)

**Fig 7:**
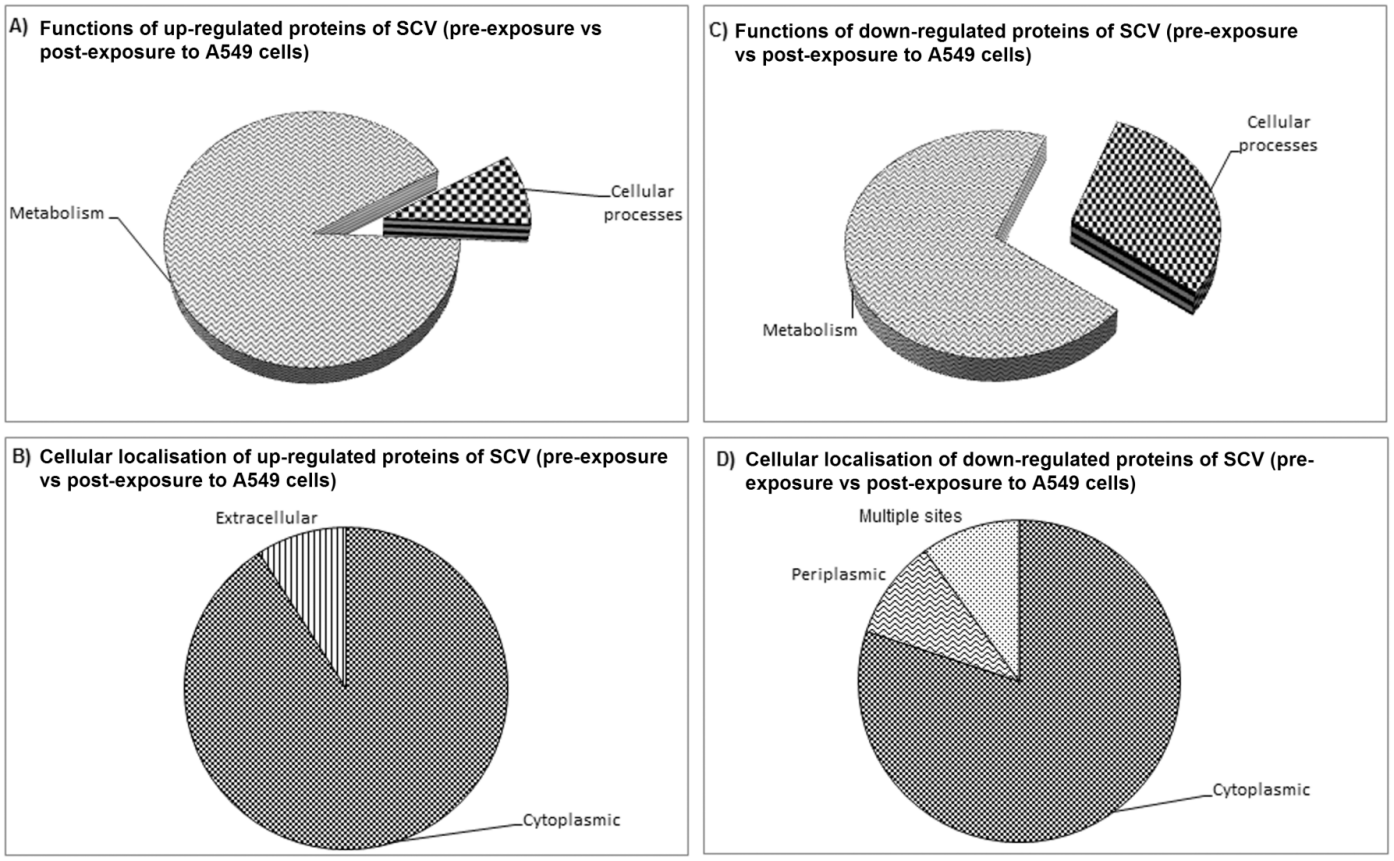
Protein categories and the subcellular localisation of *Burkholderia pseudomallei* WT (pre-exposure vs post-exposure to A549 cells). Functional protein categories were predicted using COG, while subcellular localisation was predicted using PSORT. Panels A and B refer to functions and cellular location of the up-regulated proteins, respectively. Panels C and D refer to functions and cellular location of the down-regulated proteins, respectively.

Among the up-regulated proteins that were detected in the SCV post-exposure comparing with SCV pre-exposure to A549 cells, 10 proteins (Eno, CbbA, Fpr, BPSL2288, HmpA, AhcY, PaaA, DapD, ArcB and Ndk) were implicated in metabolism of the bacteria and one (FliC) in cellular processes (cell motility) (Table 4; Fig 7A). However, localisation prediction revealed that 90.9% were of cytoplasmic and 9.1% of extracellular (Fig 7B). Whereas, among the down-regulated proteins, seven (BkdA2, ScoB, ScoA, PhbA, Adk, ArgT, and BPSL1167) were involved in metabolism and three in cellular processes (BPSS2288, GroEL1 (n = 5) and BPSL2748) (Fig 7C). Of the 10 down-regulated proteins, 80% were of cytoplasmic origin, 10% of periplasmic and 1% of multiple localisation sites (Fig 7D).

ArgT was the only protein predicted to contain signal sequence cleavage sites secreted via the classical sec-pathway using the SignalP version 4.0.

## Discussion

Despite antimicrobial therapy, *B*. *pseudomallei* infections account for up to 20% of community acquired septicemias and causes a significant number of deaths in up to 40% of treated patients in endemic regions of the world [27, 28]. The onset of this disease may be attributed to the presence of specific virulence factors of this pathogen [13] including type IV pili [29], exopolysaccharide capsule [30, 31], lipopolysaccharide O antigen [32], and type II, III, and VI secretion systems [33–35]. Some of these virulence factors play important roles in adherence, invasion or disruption of host cell membranes and therefore, may facilitate intracellular survival and persistence in host cells or cell-to-cell spread [13, 27]. Recent studies have also associated disease pathogenesis with variations in bacterial morphology [16, 36–38]http://www.sciencedirect.com/science/article/pii/S1874391914001729-bb0105. SCVs of other pathogenic bacteria have been reported to facilitate their survival in stress and harsh environment by adapting their size [39] and thus, protecting themselves from host defenses and antibiotics compared with their WT [1, 10, 40]. In *B*. *pseudomallei*, SCVs were found to produce more biofilms *in vitro* and exhibit significantly reduced ability to kill *Caenorhabditis elegans* relative to the WT [17]. However, the association between colony morphological variations in *B*. *pseudomallei* with intracellular survival and cytotoxicity has seldom been investigated [16].

In this study, the intracellular survival efficiency of both WT and SCV morphotypes has been assessed during interactions with human lung epithelial cells, A549. The immortalised A549 cells originating from a human alveolar cell carcinomas were used in this study since they have similar morphological and biochemical features of type II alveolar epithelial cells of the intact lung [41]. Thus, the host-pathogen interaction of *B*. *pseudomallei* and A549 cells may increase our understanding in the bacterial responses to the intact human cells during the respiratory tract infection. In addition, A549 cells are also easier to culture with a high proliferation rate and long lifespan as compared to primary cells that have limited replicative potential and may become senescent in culture [42] and lead to inconsistent results *in vitro*. Additionally, primary cells obtained from different patients may behave differently in culture conditions based on the genetics and age of patients [43]. A549 cells have frequently been used in many studies involving *Burkholderia* species to assess its effects on invasion and intracellular growth as the lung cells represent a biological target for these bacteria [20, 44–47] and are useful for comparative studies. However, the data presented here should be treated with caution since tumor cells in culture are prone to genotypic and phenotypic drifting [48, 49].

At both the MOIs of 1:10 and 1:100, between 0 to 12 hours post-infection, the WT appeared to be more effective in intracellular replication within the A549 cells than the SCV. We presume that this may be attributed to the rapid growth of the WT compared with the slow-growing SCV [18]. Interestingly, the intracellular replication of WT was reduced at the higher MOI of 1:100 following 10 hours post-infection. These data suggest that intracellular replication of *B*. *pseudomallei* in the epithelial cells is saturable as previously demonstrated in *B*. *cepacia* [50]. In contrast, the replication and intracellular survival of SCV increased between 10 and 12 hours at both MOIs which suggest that SCV might be able to survive longer in the A549 cells. To quantitate the integrity and cellular viability of the *B*. *pseudomallei* infected A549 cells, LDH release was measured in the cytotoxicity assays for both WT and SCV morphotypes. Cytotoxicity assays showed that SCV caused the A549 cells to release less LDH compared with the WT, and were less cell cytotoxic at 12 hours post-infection at both the MOIs. However this difference is not significant. The association between morphology variations in *B*. *pseudomallei* with intracellular survival in epithelial cells and macrophages have demonstrated by Tandhavanant *et al*., whereby the different morphotypes were found to be associated with alteration in the ability of to survive in A549 cells and mouse macrophage cell line J774A.1 [16]. In agreement with this, several reports have also described the association between SCV and persistence in other pathogenic bacteria. Roggenkamp *et al*., suggest that the persistence of bacterial infections are typically associated with slow-growing microorganisms and correlated with the appearance of SCVs [51]. Others, have also described similar correlation in other pathogens, such as *Salmonella*, in which the SCV isolates of *Salmonella enterica* serovar Typhimurium were associated with long-term persistence in fibroblasts [52]. Additionally, in *E*. *coli*, the SCVs have also been associated with persistence [51] and in *S*. *aureus*, it has been demonstrated that the intracellular survival and antibiotic resistance facilitate the persistence of SCV [36, 53]. Moreover, Proctor *et al*., [1, 54] proposed that the phenotypic characteristics of SCVs of *S*. *aureus* were associated with persistent infection.

Several reports have also described the importance of persistence in *B*. *pseudomallei* infections. Goodyear *et al*., suggest that efficient dissemination of *B*. *pseudomallei* to other organs may require persistent *B*. *pseudomallei* with lower-level infection [55], while others suggest that the persistent *B*. *pseudomallei* was found to be resistance to the drugs effective against aerobic *B*. *pseudomallei* and to drugs that target anaerobic bacteria [56]. In addition others also demonstrated that recurrent infection result from relapse are due to failure to clear the *B*. *pseudomallei* [57, 58]. Thus, the ability of *B*. *pseudomallei* SCV to survive longer in host cells could render the infection difficult to be eradicated and could also be one of the reasons for the high recurrent infection in melioidosis. The association of SCV with recurrent infection and persistence has been described previously [1]. Haussler *et al*., have described the difficulty to eradicate *B*. *pseudomallei* SCV in a mouse model of melioidosis despite proper antibiotics treatment [59]. In addition, the capability of *B*. *pseudomallei* SCV to survive longer in host cells could be due to adaptation in the bacterial cells to support its persistence including morphology switching to SCV [1, 59] or due to the wide regulatory alterations in host cells supporting bacterial persistence, including virulence and immunogenic factors inactivation and deletion of environmental survival pathways [60].

The correlation between morphological variations in *B*. *pseudomallei* with pathogenesis has not been extensively investigated. The present study was therefore conducted to identify the differences in proteins profiles of mid-log phase *B*. *pseudomallei* WT and SCV, induced after exposure to A549 cells for two hours. Several studies have demonstrated the changes in the expression of bacterial proteins upon exposure to environmental changes [61–63] but, to our knowledge, this is the first report on profiling and identification of the differences in the expression of *B*. *pseudomallei* proteins post-exposure to A549 cells. Moreover, the differentially expressed proteins of both WT and SCV post-exposed to the A549 were compared with those of both variants under the pre-exposed conditions.

### WT vs SCV (post-exposure to A549 cells)

In our previous study, adherence of SCV were found to be significantly higher as compared with the WT [18]. Similarly in another study, the SCVs of *P*. *aeruginosa* were also reported to have higher efficiency of adherence to epithelial cells as compared with the WT [64]. Several proteins (*i*.*e*. GapA, CbbA and BetB) observed to be up-regulated in SCV vs WT (post-exposure to A549 cells) may support the finding that SCVs are more adherent than the WT. These proteins have been associated with adhesion and virulence in other pathogenic bacteria [65–68]. Mutation of *GapA-1* of *Neisseria meningitides* has been reported to significantly affect the ability of this organism to adhere to human epithelial and endothelial cells [65]. GAPDH enzyme (encoded by *GapA* gene) has been reported to play a role in adherence to Caco-2 cells in *Lactobacillus plantarum*, and to gastric mucin in both *L*. *plantarum and Mycoplasma genitalium* [66, 67, 69]. In a study on *N*. *meningitides*, *CbbA* mutant was found to demonstrate a significant reduction in the adhesion to HBME and 36 HEp-2 cells compared with its isogenic parent [68]. *Brucella abortus betB* mutant was reported to be unable to replicate in HeLa and RAW 264.7 macrophages cells, whereas in mice, the *betB* mutant was cleared from the spleen compared with the wild-type strain [70]. In this study, the up-regulation of GapA, CbbA and BetB in SCV post-exposure to cells suggest that these proteins may have played a role in the higher efficiency of SCV adherence to, and prolong intracellular survival, in A549 cells as compared with WT.

Additionally, the Mdh, BetB, SucD and BPSL2288, were also found to be up-regulated in the SCV post-exposure as compared with the WT post-exposure to A549 cells, and these proteins have been reported to be associated with energy production [71–74]. We postulate that the up-regulation of these proteins in SCV post-exposure compared with the WT post-exposure may be due to the high efficiency of SCV to adhere to A549 cells as suggested in another study on *Neisseria gonorrhoeae*, where energy was demonstrated to be required for maximal adherence of the bacteria to phagocytic and nonphagocytic cells [75].

Eno, among the down-regulated proteins in SCV post-exposure as compared with the WT post-exposure, has been reported to play a role in invasion and virulence in other pathogenic bacteria [76–79]. The Eno protein has been associated with invasion, pathogenesis and spread of bacteria from one cell to another causing systemic infection in *Bacillus anthracis*, *Streptococcus pneumoniae*, *Aeromonas hydrophila* and could be used as a potential vaccine candidate in other pathogenic bacteria [76–78, 80, 81]. The down-regulation of Eno in SCV post-exposure to A549 cells (up-regulated in WT) supports our finding in a recent study, in which the WT was demonstrated to be more efficient in invasion and cell-to-cell spreading than SCV [18].

In addition, ScoA and ScoB were also down-regulated in the SCV post-exposure to A549 cells. ScoA and ScoB were reported to be co-transcribed as a single mRNA, furthermore ScoA was also found to be down-regulated upon exposure to exogenous oxidative stress in *B*. *pseudomallei* [82] which may be used by the cell to kill the bacteria [83]. Down-regulation of ScoA and ScoB in SCV may suggest that SCV has a higher ability to resist the exogenous oxidative stress compared with the WT. Two other proteins, FtsZ and ArgT were also found to be down-regulated in SCV post-exposure. FtsZ is a well-known protein that plays an essential role in cell division [84, 85] and *S*. *typhimurium argT* mutant was reported to be attenuated in growth in both the macrophages and the mice model of infection [86]. Down-regulation of these proteins in SCV post-exposure (up-regulated in WT) may suggest that the SCV is less active in multiplication which is in line with our recent finding where it was found that the SCV multiplied less actively compared with the SCV [18].

### WT (pre-exposure vs post-exposure to A549 cells)

Among the up-regulated proteins in WT post-exposure as compared with the WT pre-exposure to A549 cells, five (*i*.*e*. Eno, Ndk, BipD, Dps and PpiB) proteins were detected and associated with virulence and pathogenesis in other pathogens and the role of these proteins, mainly in invasion, as well as in adhesion, development of systemic infection and intracellular survival had been previously reported. The Eno protein has been linked to virulence, invasion, metastasis, and development of systemic infection in other pathogens as mentioned earlier [76–78]. In *Mycobacterium tuberculosis*, Ndk was demonstrated as a virulence factor due to its ability to inhibit phagosome maturation in murine RAW 264.7 macrophages and promote survival and persistence of mycobacteria within the macrophage [87]. The BipD protein is an invasion protein associated with the Type III secretion system of *B*. *pseudomallei* [88]. A *BipD* mutant in *B*. *pseudomallei* was reported to have reduced intracellular survival in J774.2 murine macrophage-like cells and also cause remarkable reduction in actin tail formation. In addition, the *BipD* mutant was also found to inhibit invasion of HeLa cells [89]. In the initial infection of a cell, BipD was reported to assist in the escape of *B*. *pseudomallei* from endocytic vesicles and is also involved in the actin polymerisation which in turn facilitates escape of the bacteria into adjacent host cells [90]. Mutants of *BipD* (in *Burkholderia*), and its homologue *IpaD* zin *Shigella*) and *SipD* (in *Salmonella*) were demonstrated to be reduced in invasiveness as compared with their parental strains [88, 91, 92]. In addition, Dps protein has been reported to provide protection *against* oxidative stress encountered during infection in *Salmonella* [93, 94], *Agrobacterium tumefaciens* and *Mycobacterium smegmatis* [95, 96] as well as *E*. *coli* [93, 97–100]. The cyclophilin B (PpiB) was also found to be associated with survival and virulence in other pathogenic bacteria. The *B*. *abortus* cyclophilins *cypAB* mutant was more sensitive to environmental stress, such as oxidative stress, pH, and detergents. In addition, the mutant strain also displays reduced virulence in BALB/c mice and defective intracellular survival in HeLa cells [101]. Therefore, the up-regulation of these proteins WT post-exposure to the A549 cells, either individually or in combination, may suggest important roles in facilitating invasion as well as the virulence and pathogenesis of *B*. *pseudomallei* in general.

Several other genes were also found to be up-regulated in WT post-exposure comparing with WT pre-exposure and have been associated with survival in other pathogenic bacteria including Fpr, ArgT and DapD. In *Pseudomonas putida*, the growth rate of a *fpr* mutant was found to be significantly reduced in the wild type [102]. Furthermore, the *fprA* gene was found to be induced by oxidative stress [102–104]. Similar to the *fpr* mutant, the growth of *argT* mutant of *S*. *typhimurium* was found to be reduced in macrophages and BALB/c mice [86]. Furthermore, the Fpr is a redox protein and previously, other redox proteins have been suggested to have the tendency to aggregate (due to their instability) or association with other proteins of the host cells [105–108] and thus, facilitate toxicity [109, 110], or cause an oxidative modification to the protein by altering the structure and function of the proteins [111–113]. Thus, these proteins may disturb the normal functions of cells and play an important role in disease pathogenicity [114, 115]. DapD is part of the L-lysine biosynthetic pathway which is crucial for the survival of the pathogen *M*. *tuberculosis* [116–118]. The up-regulation of these proteins in WT post-exposure may suggest their important roles in the survival and active growth comparing with WT pre-exposure to A549.

In addition, the proteins GapA and CbbA were found to be down-regulated in the WT post-exposure to A549 cells. These proteins have been reported to be associated with adhesion [65–69]. Down-regulation of these proteins in the WT post-exposure in the line with low adherence efficiency observed in the WT [18] and also with our finding in the SCV post-exposure compared with WT post- exposure to A549, whereby there was an up-regulation of these two proteins in the SCV post-exposure. Additionally, in our previous study we have convincingly demonstrated that the SCV is more adherent than the WT [18].

### SCV (pre-exposure vs post-exposure to A549 cells)

Among the up-regulated proteins in SCV post-exposure when compared with SCV pre- exposure to A549 cells, the proteins CbbA, Eno, Ndk, FliC and PaaA were identified and found to play roles mainly in bacterial adhesion, invasion, intracellular survival and persistence and virulence in pathogenic bacteria including *B*. *pseudomallei*. The Eno, Ndk and CbbA proteins play a role in adhesion, invasion, virulence, survival and persistence and pathogenesis of bacteria as mentioned earlier [68, 76–78, 87]. Flagellin (FliC) is a surface-associated protein in *B*. *pseudomallei* that is required for motility [119, 120]. Motility has been demonstrated as one of the virulence factors in many bacteria [121–124]. In *B*. *pseudomallei*, FliC was reported to facilitate invasion into non-phagocytic cells [125] and *fliC* mutant leads the bacteria to become avirulent during intranasal infection of mice and these mice remained healthy and did not show any symptom of disease [124]. However, FliC in *E*. *coli* has been reported to be required for adherence and invasion [126–128] while, in of *Clostridium difficile*, it has been found to enhance gut colonisation and persistence [129]. In *E*. *coli O157*, FliC was reported to be required in persistence and colonisation in chickens [130]. In addition, the virulence of *Burkholderia cenocepacia paaA* mutant was found to be reduced compared with the WT type in *C*. *elegans* model of infection [131].

Several other proteins which were also found to be up-regulated in SCV post-exposure compared with SCV pre-exposure include Fpr, DapD, HmpA and ArcB. The roles of the Fpr and DapD proteins in the growth and survival in other pathogenic bacteria have been described previously [102–104, 116–118]. However, HmpA was described to be involved in detoxification of nitric oxide [132] and the *hmp* mutant of *E*. *coli* demonstrated lesser survivability compared with the wild-type bacteria in macrophages [133]. Thus, it was suggested that the Hmp confer resistance to killing within the macrophages [133]. Activity of HmpA in *Salmonella*-infected macrophages was also reported to affect bacterial viability [134, 135]. The ArcB protein is known to be regulated by the arginine deiminase pathway that is implicated in amino acid transport and metabolism. In *P*. *aeruginosa*, this pathway is induced under anaerobic conditions stress for ATP synthesis [136].Thus, the up-regulation of these proteins especially HmpA and ArcB which were only found to be up-regulated in the SCV post-exposure to A549 cells is suggestive of the role of these proteins in the intracellular survival of the SCV, in support of our finding that SCV can survive longer than WT in A549 cells.

On the other hand, several proteins were found to be down-regulated in the SCV post-exposure comparing with SCV pre-exposure to A549 cells. These include ScoA, ScoB and ArgT. ScoA was down-regulated in *B*. *pseudomallei* subjected to oxidative stress and co-transcribed with ScoB as a single mRNA as discussed earlier [82]. The down-regulation of ScoA and ScoB in SCV may be attributed to resistance towards clearance by the extracellular antioxidant enzymes of the cells [83], thus, allowing it to survive more efficiently inside the host cells.

Predictions of cellular localisation using PSORT analyses showed that majority of the differentially expressed proteins (in all three comparisons including WT vs SCV (post-exposure to A549 cells), WT (pre-exposure vs post-exposure to A549 cells) and SCV (pre-exposure vs post-exposure to A549 cells)) identified in both the WT and SCV were originated from the cytoplasm. This is not unusual since similar findings have been reported in other bacteria including *B*. *pseudomallei* [137], *Erwinia chrysanthemi* [138] and *B*. *cepacia* [47]. Active cell division of bacteria during the mid-logarithmic phase of growth, can explain the presence of more than 60% of up-regulated and down-regulated proteins implicated in metabolic pathways and required for colonisation of bacteria [47].

Among the differentially expressed proteins, several isoforms were demonstrated including GroEL1 isoforms (n = 8), in WT post-exposure vs WT pre-exposure whereas BkdA2 isoforms (n = 2) and GroEL1 isoforms (n = 5) were found in SCV post-exposure vs SCV pre-exposure to A549 cells. Similar findings have been reported previously in *B*. *pseudomallei* and other pathogenic bacteria [139–143] which may be due to post-translational modifications during bacterial multiplication including phosphorylation, glycosylation, methylation, deamidation, and biotinylation, each of which can influence the charge and the isoelectric point [140]. Most eukaryotic protein isoforms will likely have the same basic function, however, some also were reported to show differences in function, sub-cellular localisation or loss of a regulatory sub-domain [144–151]. However, these differences in function, sub-cellular localisation or loss of a regulatory sub-domain have been seldom studied in bacteria and/or SCV. Thus, further investigation is warranted in order to elucidate these differences in bacteria.

## Conclusion

This study has shown that SCV of *B*. *pseudomallei* may have the ability to survive longer in A549 epithelial cells compared to the WT. Thus, we hypothesise that the SCVs may be among the strategy of *B*. *pseudomallei* to facilitate persistence. Furthermore, the 2D-GE approaches led to the prediction of several *B*. *pseudomallei* determinants which may play important role in or associated with pathogenesis. The differences observed in the expressed proteins underline the association of these proteins with the morphotypic and phenotypic changes of colony morphology variants. These include several proteins that may be implicated in virulence or pathogenesis and may reflect the differences observed in the ability of each of the variants to adhere, invade, survive and persist in human and animal cells. This may in turn expand our knowledge on pathogenesis of melioidosis with regards to antibiotic resistance, recurrence and the aggressiveness of the disease.
